# Diagnostic Revision From Primary Lateral Sclerosis to Amyotrophic Lateral Sclerosis: A Cohort Study

**DOI:** 10.1212/WNL.0000000000218055

**Published:** 2026-05-27

**Authors:** Eva M.J. de Boer, Sean W. Willemse, Jan H. Veldink, H. Stephan Goedee, Alexander F.J.E. Vrancken, Wouter van Rheenen, Henk-Jan Westeneng, Leonard H. van den Berg, Michael A. van Es

**Affiliations:** From the Department of Neurology, Brain Center Rudolf Magnus, University Medical Center Utrecht, the Netherlands.

## Abstract

**Background and Objectives:**

Primary lateral sclerosis (PLS) is defined as a pure upper motor neuron syndrome and is a diagnosis of exclusion, amyotrophic lateral sclerosis (ALS) being the most likely alternative diagnostic consideration. A minimum disease duration of 2 years is required for the diagnosis of PLS, after which patients are classified as probable PLS (P-PLS) and subsequently as definite PLS (D-PLS) after 4 years. Our aim is to apply the current diagnostic criteria to a population-based cohort and investigate which clinical characteristics are associated with a diagnostic revision to ALS.

**Methods:**

This cohort study included patients meeting the current diagnostic criteria for PLS retrospectively from the Dutch Motor Neuron Disease Registry. Diagnostic revision to ALS was based on clinical assessment, EMG findings according to the revised El Escorial Criteria, or if patients had died from disease progression within 4 years of disease onset. Clinical characteristics were compared for patients who underwent diagnostic revision with ALS vs true PLS. Subdistribution hazard ratios (SHRs) for characteristics associated with diagnostic revision were determined using Fine-Gray regression.

**Results:**

We included 478 patients (median age of onset 59.3 years, interquartile range 50.8–67.0, 47.9% female), of whom 311 (65.1%) met criteria for P-PLS and 167 (34.9%) for D-PLS at diagnosis. Eighty-eight patients (18%) underwent diagnostic revision to ALS, 76 cases (86%) before 4 years of disease duration. Patients whose diagnosis was revised to ALS had higher median age at onset (63.4 vs 58.0 years, *p* = 5.20 × 10^−4^), more often had bulbar onset (38.6% vs 19.7%, *p* = 6.19 × 10^−4^), and faster progression (median ALS Functional Rating Scale-revised slope 0.43 vs 0.18, *p* = 6.05 × 10^−11^). The risk of diagnostic revision increased if progression rate was faster (SHR 3.08 95% CI 1.69–5.60, *p* = 2.35 × 10^−4^) and if diagnosis was P-PLS compared with D-PLS (SHR 3.08, 95% CI 1.65–5.74, *p* = 3.96 × 10^−4^).

**Discussion:**

In our cohort, most diagnostic revisions from PLS to ALS were in patients with a disease duration of less than 4 years. Besides disease duration, a faster progression rate was associated with diagnostic revision from PLS to ALS. Adding progression rate to the current diagnostic criteria could increase accuracy and help identify patients at higher risk of developing ALS.

## Introduction

Primary lateral sclerosis (PLS) is a rare neurodegenerative disorder that primarily affects upper motor neurons (UMNs), leading to spasticity, loss of mobility, and mild weakness. It is highly disabling, but not necessarily life-shortening, and survival is often longer than 15 years.^1,2^ PLS is diagnosed by exclusion, amyotrophic lateral sclerosis (ALS) being one of the main alternatives to be considered. ALS is a progressive neurodegenerative disorder with combined involvement of the UMN and lower motor neurons (LMNs), invariably leading to severe weakness, with a median survival of 3–5 years from symptom onset.^3^ Initially there may only be UMN symptoms; UMN-predominant ALS typically has a slower disease course than the classic variant.^4-7^ Currently, there is no diagnostic test that can definitively distinguish PLS from UMN-predominant ALS in the early stages. Therefore, clinical follow-up is necessary to repeatedly confirm the absence of LMN involvement and establish the diagnosis PLS.

Over the years, multiple diagnostic criteria for PLS have been proposed, all requiring a minimum disease duration during which the disorder must remain a pure UMN syndrome. The first set of criteria, proposed in 1945 by Stark and Moesch and based on a case series of 43 patients, required a minimum disease duration of 5 years.^8^ In 1992, the Pringle criteria shortened the required disease duration to 3 years and incorporated EMG, based on a literature review and 8 cases.^9^ In a series of 29 cases, Gordon et al.^4^ found that 13 patients developed UMN-predominant ALS within 4 years from onset and therefore proposed a minimum duration of 4 years. The current criteria (2020) were formulated to accelerate research into treatment and the basic histopathology of PLS and are consensus based. They introduced the categories “definite PLS” (D-PLS) for patients with a disease duration ≥4 years and “probable PLS” (P-PLS) for patients with a disease duration between 2 and 4 years.^10^

Several studies have applied the current consensus criteria to a longitudinal cohort. Witzel et al. observed that the diagnosis remained unchanged at follow-up (at a median disease duration of 6.1 years) in 9 patients with P-PLS and 10 patients with D-PLS. Nevertheless, longitudinal data were unavailable for a substantial proportion of cases—55% of patients presenting with P-PLS and 66.7% with D-PLS.^11^ By contrast, de Vries et al.^12^ followed a cohort of 86 patients for more than 10 years, during which time the diagnosis of 11.6% of patients with PLS was revised to ALS (2/6 P-PLS and 3/37 D-PLS patients). In a recent study by Lester et al.,^2^ 20% of a cohort of 69 patients initially diagnosed with PLS underwent diagnostic revision to ALS. However, it was not mentioned whether these patients were initially classified as P-PLS or D-PLS.

Past and current criteria have relied on small numbers of patients and/or expert opinion. Prolonged diagnostic uncertainty can be distressing, as differentiating PLS from ALS has profound prognostic consequences. It is therefore clinically highly relevant to define the appropriate disease duration to reliably diagnose PLS. We studied the clinical and electromyographical findings associated with diagnostic revision to ALS in a large population of patients meeting the current consensus PLS criteria.

## Methods

### Patients

Patients were recruited from the ongoing Dutch population-based motor neuron disease (MND) registry, which has been described previously.^13^ Patients who have been diagnosed with MND are prospectively included in a central registry, which was started in 2006. Patients are identified either in our tertiary referral center in the University Medical Center in Utrecht or through annual screenings of registries of other hospitals in the Netherlands. Data are entered into the registry by research physicians based on medical records. Survival data for all patients included in the Dutch MND registry are collected regularly from the online municipal register.

We included patients in our cohort according to the current consensus criteria for PLS.^10^ Patients were included if they were aged 25 years and over and had had an isolated UMN syndrome in 2 or 3 regions for at least 2 years after symptom onset, up to the date of censoring (July 25, 2025). As it is a historical cohort, some of the patients included did not yet have the required minimal disease duration of 2 years during the first outpatient visit or at entry in the registry but did meet this criterion during follow-up. Furthermore, patients were excluded if they fulfilled the revised El Escorial EMG criteria (rEEC) for ALS.^14^ Patients who initially presented with PLS but received an alternative diagnosis during follow-up (e.g., hereditary spastic paraplegia [HSP] or myelopathy) were also excluded from the cohort.

### Data Collection

Baseline characteristics were collected at diagnosis or within a 3-month window after diagnosis and included sex, age at onset, disease duration (time from perceived symptom onset to death or to the date of censoring), site of onset (bulbar, spinal in arms or legs or generalized if symptom onset was in multiple regions at once), regions of UMN involvement at diagnosis (bulbar, cervical, lumbosacral), *C9orf72* status, and percentage of predicted forced vital capacity (FVC) relative to reference standard.^15-17^ Progression rate was defined as the slope (decrease per month) on the revised ALS Functional Rating Scale-revised (ALSFRS-R) total score between onset and diagnosis.^18^ In addition, concentric needle EMG data were extracted from the patients' clinical records. EMG findings were classified as normal, denervation, reinnervation, or combined denervation and reinnervation. Denervation was defined as fibrillations and/or positive sharp waves and/or complex repetitive discharges. Reinnervation was defined as large polyphasic motor unit potentials and/or giant potentials. Findings were collected for each of the examined regions (bulbar, cervical, thoracic, and lumbosacral). Furthermore, the number of regions with any abnormal finding was collected. Patients were excluded if they had EMG findings in any region meeting the rEEC for ALS at any time point before a disease duration of 2 years was reached.^14,19^

### Outcomes

We revised the diagnosis of PLS to ALS based on clinical assessments (finding of atrophy and/or weakness during neurologic examination) and/or EMG findings fulfilling the rEEC for ALS in one or more anatomic regions (bulbar, cervical, thoracic, and/or lumbosacral) or if the patient died before reaching a 4-year disease duration, as this is highly atypical for PLS and more fitting for ALS. As survival status is routinely collected in our registry, but cause of death is not, the medical records of patients who passed away before a disease duration of 4 years were checked and individuals were excluded if cause of death was not due to disease progression (e.g., euthanasia).

### Statistical Analysis

Survival and time from disease onset to diagnostic revision to ALS were reported using Kaplan-Meier estimates as a median with 95% CI. The median survival was compared using a log-rank test. Clinical and EMG characteristics of patients with diagnostic revision to ALS and true PLS were compared using a Mann-Whitney *U* test, χ^2^ test, or Fisher exact test where applicable. To determine which clinical characteristics contribute to a higher chance of diagnostic revision to ALS, Fine-Gray regression analysis was performed to account for the competing event of all-cause mortality after 4 years. Patients entered the model at a disease duration of 2 years after the date of onset. Because of missing data, multiple imputation was performed with 100 iterations using the smcfcs package in R (version 4.3.2), which imputed data specifically for use in a Fine-Gray regression model.^20^ Model estimates were pooled using Rubin rules and presented as subdistribution hazard ratios (SHRs) with 95% CI.^21,22^ *p* Values of 0.05 and lower were deemed statistically significant.

### Standard Protocol Approvals, Registrations, and Patient Consents

The Dutch MND Registry, of which this study is a part, has been approved by the medical-ethical committee (NedMec) of the University Medical Center Utrecht. All patients described in this article have given written informed consent for the use of their data for research purposes.

### Data Availability

Anonymized data not published within this article will be made available by request from any qualified investigator.

## Results

We included 478 patients of whom 311 (65.1%) initially met the criteria for P-PLS and 167 (34.9%) met the criteria for D-PLS (Table 1). Of the patients meeting the criteria for P-PLS, 51.4% presented with a disease duration shorter than 2 years. In 88 patients (18.4% of the cohort), diagnosis was revised from PLS to ALS; that is, 24.4% of the P-PLS group (n = 76, 86.4% of diagnostic revisions) compared with 7.2% (n = 12, 13.6% of diagnostic revisions) of D-PLS. Forty-seven (53.4%) patients underwent diagnostic revision based on clinical and electrophysiologic signs, while in 31 (35.2%) patients, this was due to death before a disease duration of 4 years had been reached. In 10 patients (11.4%), the diagnosis was revised based on EMG abnormalities fulfilling the rEEC without overt clinical signs of LMN dysfunction. Diagnosis was more frequently revised in female patients (59.1% vs 40.9%, *p* = 0.020) and in those with a higher age at onset (median 63.4 years vs 58.0 years, *p* = 5.20 × 10^−4^). Diagnostic revision was more frequent in patients with a bulbar onset and less frequent in patients with onset in the lower limbs (*p* = 6.19 × 10^−4^). The rate of progression measured by the ALSFRS-R slope was faster for patients who underwent diagnostic revision compared with those whose eventual diagnosis was PLS (median 0.43 vs 0.18 points decrease per month, *p* = 6.05 × 10^−11^).

**Table 1:** Clinical Characteristics of the Total Cohort, According to Eventual Diagnosis

	Total cohort	Diagnostic revision to ALS	True PLS	*p* Value
No. of patients	478	88	390	—
Sex, no. of men, n (%)	249 (52.1)	36 (40.9)	213 (54.6)	0.020
Deceased at time of data extraction, n (%)	237 (49.6)	73 (83)	164 (42.1)	4.15 × 10^−12^
Age at onset, y, median (IQR)	59.3 (50.8–67.0)	63.4 (54.7–69.7)	58.0 (49.8–66.3)	5.20 × 10^−4^
Survival since onset, mo, median (95% CI)	218 (188–237)	63.3 (45.7–83.6)	237 (222–277)	2.0 × 10^−16^
Conversion type, n (%)			—	—
Clinical and EMG	47 (53.4)	47 (53.4)		
EMG only	10 (11.4)	10 (11.4)		
Death before 4 y	31 (35.2)	31 (35.2)		
Disease duration to conversion, mo, median (95% CI)	42.9 (38.2–45.2)	42.9 (38.2–45.2)	—	—
Disease duration at baseline, n (%)				5.36 × 10^−9^
Disease duration <2 y at presentation	160 (33.5)	53 (60.2)	107 (27.4)	
Disease duration 2–4 y at presentation	151 (31.6)	23 (26.1)	128 (32.8)	
Disease duration ≥4 y at presentation	167 (34.9)	12 (13.6)	155 (39.7)	
2020 consensus criteria category at study entry				3.47 × 10^−6^
Probable PLS	311 (65.1)	76 (86.4)	235 (60.3)	
Definite PLS	167 (34.9)	12 (13.6)	155 (39.7)	
Site of onset, n (%)				6.19 × 10^−4^
Bulbar	111 (23.2)	34 (38.6)	77 (19.7)	
Spinal	358 (74.9)	54 (61.4)	304 (77.9)	
Arms	32 (6.7)	10 (11.4)	22 (5.6)	
Legs	326 (68.2)	44 (50.0)	282 (72.3)	
Generalized	9 (1.9)	0 (0)	9 (2.3)	
No. of regions^a^ UMN involvement, n (%)				0.034
2	217 (45.4)	31 (35.2)	186 (47.7)	
3	261 (54.6)	57 (64.8)	204 (52.3)	
*C9ORF72* tested n (%)	257 (53.8)	52 (59.1)	205 (52.6)	0.099
*C9ORF72 *mutated, n (%)	5 (1.0)	2 (2.3)	3 (0.8)	
FVC % of predicted at baseline, median (IQR)	94.0 (83.8–105.0)	94.5 (82.0–108.3)	93.5 (84.0–104.0)	1
Missing (%)	210 (43.9)	32 (36.4)	178 (46.5)	
ALSFRS-R total score at baseline, median (IQR)	38.0 (34.0–42.0)	38.0 (32.5–41.0)	38.0 (34.0–42.0)	0.387
ALSFRS-R slope median (IQR)	0.20 (0.10–0.40)	0.43 (0.24–0.62)	0.18 (0.09–0.33)	6.05 × 10^−11^
Missing (%)	110 (23.0)	25 (28.4)	85 (21.8)	

Abbreviations: ALS = amyotrophic lateral sclerosis; ALSFRS-R = ALS Functional Rating Scale (slope = [48 − ALSFRS-R score]/[date of the ALSFRS-R score minus date of onset in months]); FVC = forced vital capacity (percentage of predicted based on normative values for age, sex, and body height); IQR = interquartile range; PLS = primary lateral sclerosis; UMN = upper motor neuron.

a Regions: lower extremities, upper extremities, bulbar.

Table 2 presents the characteristics of the 311 patients who were within the P-PLS category at the time of study entry and categorized as true PLS (n = 235) or revision to ALS (n = 76). Of the patients from the P-PLS category whose diagnosis was revised before a disease duration of 4 years, revision was based on clinical symptoms and EMG findings in 38 (50%), on EMG findings solely in 7 (9.2%), and in 31 (40.8%) cases, it was due to death before reaching a 4-year disease duration. Patients who underwent diagnostic revision before reaching a disease duration of 4 years were more frequently female (65.8% vs 43.7%, *p* = 3.26 × 10^−3^), had a higher median age at onset (64.4 vs 59.5 years, *p* = 3.54 × 10^−3^), a shorter disease duration at presentation (69.7% presented before 2 years vs 45.5%, *p* = 2.43 × 10^−4^), and a higher median ALSFRS-R slope (0.49 vs 0.26, *p* = 4.06 × 10^−6^), compared with patients with true PLS who presented with a symptom duration of less than 4 years. Furthermore, the median survival since onset was markedly shorter for patients with diagnostic revision to ALS (46.9 months, 95% CI 42.6–68.1 vs 204, 95% CI 180–233, *p* < 2.0 × 10^−16^).

**Table 2:** Clinical Characteristics of Patients Presenting Before a 4-Year Disease Duration, According to Eventual Diagnosis

	Total	Diagnostic revision to ALS	True PLS	*p* Value
No. of patients	311	76	235	—
Sex, no. of men, n (%)	152 (48.9)	26 (34.2)	126 (56.3)	3.26 × 10^−3^
Deceased at time of data extraction, n (%)	162 (52.1)	67 (88.2)	95 (40.4)	4.46 × 10^−13^
Age at onset, y, median (IQR)	60.8 (52.6–68.7)	64.4 (56.0–72.0)	59.5 (52.1–67.9)	3.54 × 10^−3^
Survival since onset, mo, median (95% CI)	163 (130–189)	46.9 (42.6–68.1)	204 (180–233)	<2.0 × 10^−16^
Conversion type, n (%)			—	—
Clinical and EMG	38 (50)	38 (50)		
EMG only	7 (9.2)	7 (9.2)		
Death before 4 y	31 (40.8)	31 (40.8)		
Disease duration to conversion, mo, median (95% CI)	40.5 (37.1–43.5)	40.5 (37.1–43.5)	—	—
Disease duration at baseline, n (%)				2.43 × 10^−4^
Disease duration <2 y at presentation	160 (51.4)	53 (69.7)	107 (45.5)	
Disease duration 2–4 y at presentation	151 (48.7)	23 (30.3)	128 (54.7)	
Site of onset, n (%)				1.72 × 10^−3^
Bulbar	88 (28.3)	33 (43.4)	55 (23.4)	
Spinal	215 (69.1)	43 (56.6)	172 (73.2)	
Arms	25 (8.0)	10 (13.2)	15 (6.4)	
Legs	191 (61.4)	33 (43.4)	158 (67.2)	
Generalized	8 (2.6)	0 (0)	8 (3.4)	
No. of regions^a^ UMN involvement, n (%)				0.085
2	138 (44.4)	27 (35.5)	111 (47.2)	
3	173 (55.6)	49 (64.5)	124 (52.8)	
*C9ORF72 *tested n (%)	164 (52.9)	44 (57.9)	120 (51.3)	0.058
*C9ORF72 *mutated, n (%)	3 (1.0)	2 (2.6)	1 (0.4)	
FVC % of predicted at baseline, median (IQR)	96.0 (86.0–106.0)	96.0 (83.5–109.8)	95.0 (87.0–105.0)	0.965
Missing (%)	122 (39.2)	26 (34.2)	96 (40.9)	
ALSFRS-R total score at baseline, median (IQR)	39.0 (35.0–42.0)	39.0 (35.0–42.0)	39.0 (35.0–42.0)	0.791
ALSFRS-R slope median (IQR)	0.30 (0.17–0.51)	0.49 (0.30–0.64)	0.26 (0.15–0.45)	4.06 × 10^−6^
Missing (%)	69 (22.3)	20 (26.3)	49 (20.9)	

Abbreviations: ALS = amyotrophic lateral sclerosis; ALSFRS-R = ALS Functional Rating Scale (slope = [48 − ALSFRS-R score]/[date of the ALSFRS-R score minus date of onset in months]); FVC = forced vital capacity (percentage of predicted based on normative values for age, sex, and body height); IQR = interquartile range; PLS = primary lateral sclerosis; UMN = upper motor neuron.

a Regions: lower extremities, upper extremities, bulbar.

For 62 patients (70.5%), time of diagnostic revision to ALS was within the 2- to 4-year timeframe from onset (Figure, A). The median survival for all patients with a diagnostic revision to ALS was 63.3 months (95% CI 45.7–83.6), as opposed to 237 months (95% CI 222–277) for patients with true PLS (Table 1). For patients who underwent diagnostic revision to ALS before a disease duration of 3 years (n = 29), the median survival was 34.9 months (95% CI 31.8–36.4). For patients who underwent diagnostic revision after a disease duration of between 3 and 4 years (n = 33), the median survival was 47.5 months (95% CI 45.0–73.8), and for diagnostic revision after 4 years (n = 26), the median survival was 200 months (95% CI 117–NA) (Figure, B).

**Figure:**
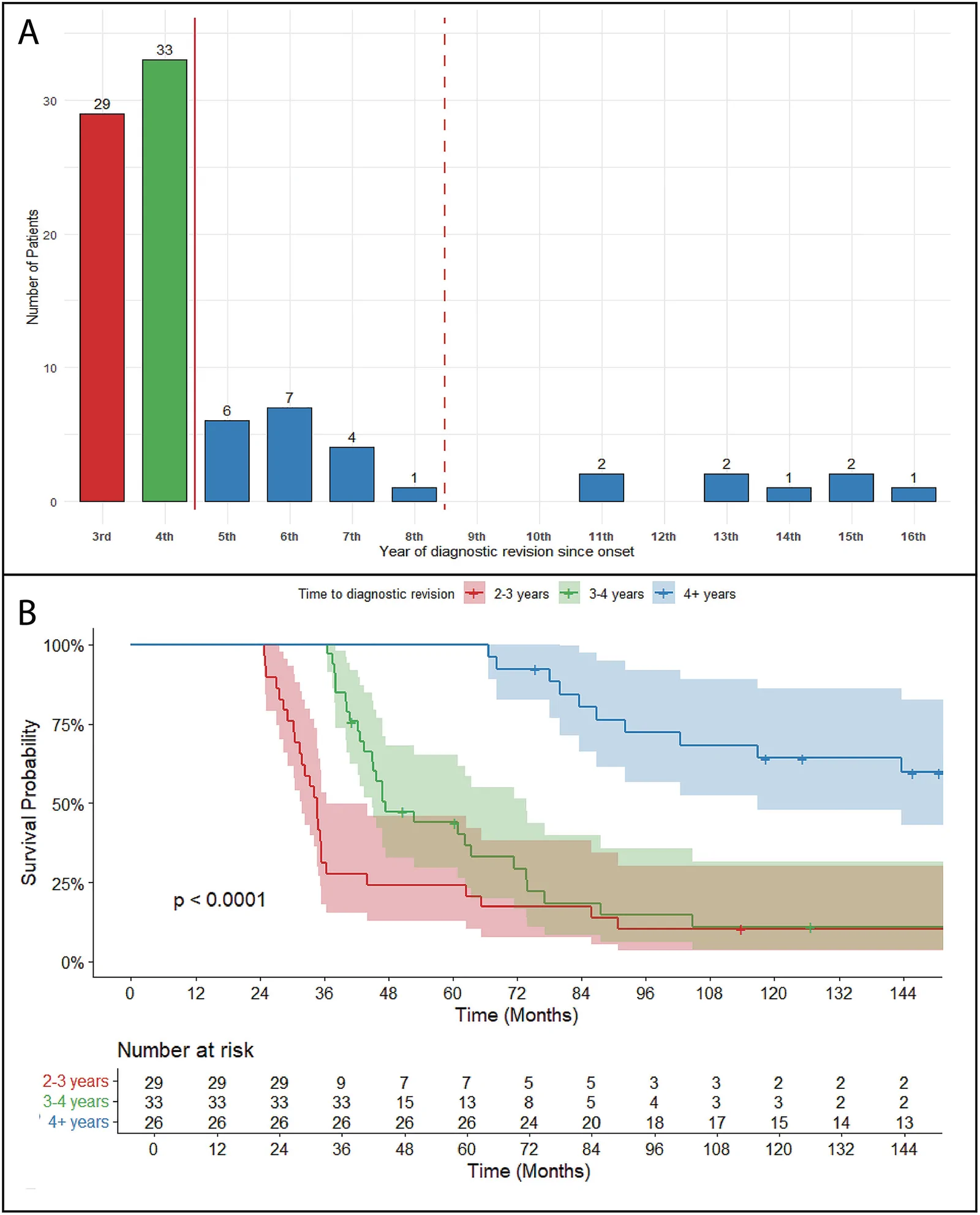
Timing of Diagnostic Revision From PLS to ALS and Associated Survival (A) All patients who underwent diagnostic revision to ALS categorized per year in which they did so after onset of symptoms. The solid line divides the P-PLS and the D-PLS groups and the number of patients who underwent diagnostic revision to ALS within the selected time frame is shown. The red dotted line represents the cut-off point where prognosis is comparable for patients who did and did not undergo diagnostic revision. (B) Survival of patients undergoing diagnostic revision categorized according to the duration from onset to year of diagnostic revision. The *p*-value represents the value of a log-rank test for the difference in median survival. ALS = amyotrophic lateral sclerosis; D-PLS = definite PLS; PLS = primary lateral sclerosis; P-PLS = probable PLS.

When comparing EMG findings (that did not meet the rEEC) for true PLS and for those whose diagnosis was revised to ALS, completely normal baseline EMG results were more frequently seen in true PLS patients (39.7% vs 27.3%, *p* = 0.013) (Table 3). When comparing regions where abnormalities were found, patients whose diagnosis was revised to ALS were found to have a greater degree of denervation or combined re- and denervation in the cervical (10.2% vs 5.2% and 10.2 vs 3.6%, respectively, *p* = 0.017) and lumbosacral (10.2% vs 4.9% and 18.2 vs 7.2%, respectively, *p* = 2.08 × 10^−3^) regions, whereas no significant differences were found for the thoracic and bulbar regions. At least 1 follow-up EMG was available for 152 (39%) patients with true PLS; in 62 of them (40.7%), the second EMG was normal, compared with 58 (65.9%) available EMGs for patients who underwent diagnostic revision to ALS, 2 of whom (3.4%) showed no abnormalities (*p* = 1.47 × 10^−7^).

**Table 3:** Concentric Needle Electromyographic Data of Patients at Baseline, According to Eventual Diagnosis

	Total	Diagnostic revision to ALS	True PLS	*p* Value
N	478	88	390	—
Baseline EMG completely normal, n (%)	179 (37.4)	24 (27.3)	155 (39.7)	0.013
Missing	64 (13.4)	10 (11.4)	54 (13.8)	
≥1 follow-up EMGs, n (%)	230 (43.9)	58 (65.9)	152 (39.0)	
First follow-up EMG normal, n (%)	64 (27.8)	2 (3.4)	62 (40.7)	1.47 × 10^−7^
Bulbar region, n (%)				0.109
Normal	348 (72.8)	61 (69.3)	287 (73.6)	
Only re-innervation	42 (8.8)	11 (12.5)	31 (7.9)	
Only denervation	1 (0.2)	0 (0)	1 (0.3)	
Re-innervation and denervation	1 (0.2)	1 (1.1)	0 (0)	
Missing	86 (18.0)	15 (17.0)	71 (18.2)	
Cervical region, n (%)				0.017
Normal	279 (58.4)	44 (50.0)	235 (60.3)	
Only re-innervation	62 (13.0)	13 (14.8)	49 (12.6)	
Only denervation	31 (6.5)	9 (10.2)	22 (5.6)	
Re-innervation and denervation	23 (4.8)	9 (10.2)	14 (3.6)	
Missing	83 (17.4)	13 (14.8)	70 (17.9)	
Thoracic region, n (%)				0.792
Normal	349 (73.0)	64 (72.7)	285 (73.1)	
Only reinnervation	29 (6.1)	7 (8.0)	22 (5.6)	
Only denervation	14 (2.9)	3 (3.4)	11 (2.8)	
Reinnervation and denervation	2 (0.4)	0 (0)	2 (0.5)	
Missing	84 (17.6)	14 (15.9)	70 (17.9)	
Lumbosacral region, n (%)				2.08 × 10^−3^
Normal	224 (46.9)	33 (37.5)	191 (49.0)	
Only reinnervation	100 (20.9)	17 (19.3)	83 (21.3)	
Only denervation	28 (5.9)	9 (10.2)	19 (4.9)	
Reinnervation and denervation	44 (9.2)	16 (18.2)	28 (7.2)	
Missing	82 (17.2)	13 (14.8)	69 (17.7)	
No. of affected regions, n (%)				0.013
None	180 (37.7)	24 (27.3)	156 (40.0)	
1	107 (22.4)	20 (22.7)	87 (22.3)	
2	68 (14.2)	22 (25.0)	46 (11.8)	
3	37 (7.7)	9 (10.2)	28 (7.2)	
4	5 (1.0)	1 (1.1)	4 (1.0)	
Missing	81 (16.9)	12 (13.6)	69 (17.7)	

Abbreviations: ALS = amyotrophic lateral sclerosis; PLS = primary lateral sclerosis.

Denervation was defined as fibrillations and/or positive sharp waves and/or complex repetitive discharges. Reinnervation was defined as large polyphasic motor unit potentials and/or giant potentials.

Using Fine-Gray regression analysis, displayed in Table 4, we found the chance of diagnostic revision to be significantly increased for the diagnostic category P-PLS compared with D-PLS (SHR 3.08, 95% CI 1.65–5.74, *p* = 3.96 × 10^−4^), and with a higher progression rate based on ALSFRS-R slope (SHR 3.08, 95% CI 1.69–5.60, *p* = 2.35 × 10^−4^). There was a lower risk of diagnostic revision to ALS for leg onset compared with any other site of onset (SHR 0.58, 95% CI 0.36–0.94, *p* = 0.026), and for a normal vs abnormal EMG at baseline (SHR 0.56, 95% CI 0.34–0.95, *p* = 0.031). Regression analysis showed no increased risk of diagnostic revision to ALS based on female vs male sex, higher age at onset, or a change in percentage of FVC at diagnosis.

**Table 4:** Subdistribution Hazard Ratios in a Competing Risk Model for Diagnostic Revision to ALS

Variable	SHR (95% CI)	*p* Value	Missing data, n (%)
Diagnosis of P-PLS vs D-PLS	3.08 (1.65–5.74)	3.96 × 10^−4^	0 (0)
Female vs male sex	1.46 (0.93–2.29)	0.100	0 (0)
Age at onset	1.01 (0.99–1.03)	0.230	0 (0)
Leg onset vs other	0.58 (0.36–0.94)	0.026	0 (0)
Normal EMG vs any EMG abnormalities at baseline	0.56 (0.34–0.95)	0.031	64 (13.4)
ALSFRS-R slope	3.08 (1.69–5.60)	2.35 × 10^−4^	110 (23.0)
% of predicted FVC at diagnosis	0.99 (0.98–1.01)	0.257	210 (43.9)

Abbreviations: ALSFRS-R slope = ALS Functional Rating Scale (slope = [48 − ALSFRS-R score]/[date of the ALSFRS-R score minus date of onset in months]); D-PLS = definite PLS; FVC = forced vital capacity; P-PLS = probable PLS; SHR = subdistribution hazard ratio.

## Discussion

In this study, we aimed to assess the frequency of diagnostic reclassification from PLS to ALS and to identify features that may help distinguish between these disorders in clinical practice. We found that the diagnosis of PLS was revised to ALS in 24.4% of the patients presenting before a disease duration of 4 years, compared with 7.2% of patients with a disease duration longer than 4 years at initial presentation. We further studied factors associated with diagnostic revision to ALS and identified P-PLS (shorter disease duration at presentation), and a faster progression rate using ALSFRS-R slope to be risk factors. Conversely, a disease onset in the legs and a normal EMG at baseline were associated with a lower risk of diagnostic revision.

In our large cohort study on PLS of 487 patients, we were able to validate the current consensus criteria, previously only performed in smaller cohorts where diagnostic revision to ALS rates ranged from 0% to 20% for all PLS cases and 0% to 33.3% for P-PLS.^11,12^ Furthermore, the population-based design with standardized data collection and long-term follow-up makes our findings generalizable and applicable in the clinical context where the diagnostic challenge of discerning PLS from ALS is relevant.

We, however, acknowledge some weaknesses in our study. As it is a population-based registry based largely on medical records, data are incomplete, especially data on functional vital capacity; this information was not collected routinely during the early years of our registry nor during the coronavirus disease 2019 pandemic. As it is a historic cohort, genetic data are not available for every patient and genetic testing was not always repeated after the discovery of new genes. As such, some patients in the true PLS group might actually have a nonrecognized diagnosis of HSP.

Furthermore, we initially added data on weight loss to our cohort, but as ∼65% was missing, we felt this basis was insufficient to allow a firm conclusion to be drawn. Finally, it would be very interesting to include data on serum neurofilament light chain, as previous research has described distinct ranges on group level for PLS and (UMN-predominant) ALS.^23-25^ Unfortunately, neurofilament data were not available for our cohort.

Although a large number of patients were seen routinely up to a disease duration of 4 years, there was no standardized schedule for the follow-up of patients included in this study. This means that there might be a bias in the follow-up data, especially whether routine EMGs were performed, as patients who deviated from a classical presentation of PLS (e.g., due to diagnostic revision) were more likely to be followed up more intensely and have an EMG more frequently (65.9% patients with diagnostic revision to ALS vs 39% true PLS). As such, it is possible that diagnostic revision events were missed or discovered later. Furthermore, given that P-PLS patients may still change diagnosis beyond the censoring date, it is plausible that our current percentage of diagnostic revision is an underestimation of the actual rate. Similarly, after a disease duration of 4 years, patients with PLS are not routinely seen in our referral center, leading to fewer follow-up EMGs for this patient group. Follow-up EMGs for true PLS patients were only performed if there were alarming symptoms raised by patients themselves or found during neurologic examination. We found that a small number of patients who underwent diagnostic revision to ALS did so due to extensive signs of LMN involvement on EMGs during follow-up without any apparent clinical LMN involvement; there could, therefore, be more patients with PLS for whom the diagnosis should be revised, but who were missed as no new EMGs were performed. However, we found that survival for patients with a late diagnostic revision to ALS was not different from those with a sustained PLS diagnosis, presumably making the clinical relevance of distinguishing a slowly progressive, UMN-predominant ALS from PLS negligible at this point in the disease course. It does, however, raise interesting questions about the classification of these diseases and to what extent LMN involvement can be accepted for a diagnosis of PLS. Pathologic and electrophysiologic involvement of LMNs in patients with clinical PLS has been described earlier.^26-28^ As such, the degree to which LMN involvement is acceptable for a diagnosis of PLS, and the timing during which this has to be reached to influence either diagnosis or prognosis requires further investigation.

Based on this study, we would propose adding red flags to the diagnostic criteria for P-PLS, including the progression rate, EMG features, and site of onset. Previously reported rates of decline range from −0.09 to −0.26, with a mean of −0.13 to −0.16 points per month on the ALSFRS-R for patients with PLS.^2,29^ Among true PLS (no diagnostic revision), we observed a median ALSFRS-R slope of −0.18 points per month. The median progression rate in our cohort for patients with diagnostic revision to ALS was a 0.43 points per month decrease in the ALSFRS-R. This is still considerably slower than progression rates typically seen in the general ALS population, which are often approximately twice as high.^30-32^ As such, we believe that progression rate is a discerning characteristic for the diagnoses of PLS and UMN-predominant ALS and could be used to differentiate between these in a clinical setting.

Although our data show differences in the presence and pattern of EMG findings between true PLS patients and patients with diagnostic revision to ALS, we believe that these differences are not sufficiently discerning to allow strict criteria to be formulated about whether involvement of certain regions or patterns of denervation and reinnervation should exclude a diagnosis of PLS based on a singular EMG. For example, we found patients with normal EMGs at baseline (27.3% of patients with diagnostic revision) that went on to develop ALS. Conversely, patients who exhibited EMG abnormalities at baseline that did not meet the rEEC remained clinically stable with an isolated UMN syndrome on neurologic examination, thereby retaining a diagnosis of PLS during long-term follow-up. Previous research with strict inclusion criteria regarding normal EMGs during the first period of follow-up yielded only true PLS diagnoses. This approach might have incorrectly led to exclusion of patients with PLS.^33^ Furthermore, progressive EMG findings in patients with PLS do not contribute to an increased risk of diagnostic revision to ALS, but they are associated with a faster disease progression.^26,27^ The true extent to which EMG findings are permissible for a diagnosis of PLS is not yet known. However, we propose that repeated EMGs are necessary to determine whether results found at baseline are consistent or progressive, as we found that the percentage of normal EMGs in the group of patients with diagnostic revision to ALS decreased dramatically from 30% to 2% after 1 additional EMG, while this percentage in true PLS remained stable at 40%. It is important to note that these findings are true at group level; they might not be applicable to each individual patient. However, for example, if a patient presents with onset in the legs, a slow progression rate, and the findings on 2 or more EMGs are normal and stable, then an early diagnosis of PLS can be discussed with more certainty than in patients with multiple incompatible clinical characteristics.

The current consensus criteria were proposed to enable earlier diagnostic certainty and to facilitate timely enrolment in (future) therapeutic trials for PLS.^34,35^ Nonetheless, further refinement appears warranted to meet these aims, as under the current criteria, patients with UMN-predominant ALS may be incorrectly diagnosed with P-PLS. This can lead to substantial heterogeneity in future PLS cohorts and trials, while simultaneously excluding patients more likely to develop ALS from appropriate ALS trials.^34,35^ The development of a prediction model for diagnostic revision to ALS, including progression rate and EMG findings, but also weight loss and serum neurofilament light chain, among other possible biomarkers, would greatly help early diagnosis of PLS. In the meantime, we recommend that clinicians exercise caution when counselling patients with a UMN-predominant disease and a duration shorter than 4 years. Particular care is warranted in cases where there is a faster progression rate or progressive EMG abnormalities on repeated needle-EMGs, as this may suggest an increased risk of subsequent diagnostic reclassification.

## Glossary

ALS: amyotrophic lateral sclerosis
ALSFRS-R: ALS Functional Rating Scale-revised
D-PLS: definite PLS
FVC: forced vital capacity
HSP: hereditary spastic paraplegia
LMN: lower motor neuron
MND: motor neuron disease
PLS: primary lateral sclerosis
P-PLS: probable PLS
rEEC: revised El Escorial EMG criteria
SHR: subdistribution hazard ratio
UMN: upper motor neuron
